# Segmentation-Based Color Channel Registration for Disparity Estimation of Dual Color-Filtered Aperture Camera

**DOI:** 10.3390/s18103174

**Published:** 2018-09-20

**Authors:** Shuxiang Song, Sangwoo Park, Joonki Paik

**Affiliations:** Department of Image, Chung-Ang University, 84 Heukseok-ro, Dongjak-gu, Seoul 06974, Korea; shuxiang0819@cau.ac.kr (S.S.); swkdn16@cau.ac.kr (S.P.)

**Keywords:** color channel registration, disparity estimation, computational camera system

## Abstract

Single-lens-based optical range finding systems were developed as an efficient, compact alternative for conventional stereo camera systems. Among various single-lens-based approaches, a multiple color-filtered aperture (MCA) system can generate disparity information among color channels, as well as normal color information. In this paper, we consider a dual color-filtered aperture (DCA) system as the most minimal version of the MCA system and present a novel inter-color image registration algorithm for disparity estimation. This proposed registration algorithm consists of three steps: (i) color channel independent feature extraction; (ii) feature-based adaptive weight disparity estimation; and (iii) color mapping matrix (CMM)-based cross-channel image registration. Experimental results show that the proposed method can not only generate an accurate disparity map, but also realize high quality cross-channel registration with a disparity prior for DCA-based range finding and color image enhancement.

## 1. Introduction

Disparity estimation is a fundamental process for three-dimensional (3D) information acquisition from an input scene. Its applications include intelligent visual surveillance, 3D scene reconstruction, robot vision and intelligent driver assistance systems, to name a few. Over the past few decades, various approaches have been proposed to obtain the disparity information. Stereo imaging is one of the most well-known methods for disparity acquisition [1]. Despite its popularity, a number of inherent limitations exist such as: (i) the high cost and volume of multiple camera systems; and (ii) missing disparity near the boundary between foreground and background regions. A time-of-flight (ToF) system is another popular method, which measures the distance using the location of a light source and the time-of-flight [2,3]. Although it has a decent measurement speed, the accuracy is easily affected by ambient light. The imaging system using structured light can provide highly accurate disparity estimation at the cost of additional illumination sources [4]. An alternative method is to estimate disparity from multiply-defocused images acquired with different focus settings. It is impractical for real-time processing because of the multiple image acquisition process using a single, fixed camera [5].

Lee et al. presented a novel computational imaging system for disparity estimation by inserting a dual color-filtered aperture (DCA) into an ordinary single camera [6]. The DCA camera generates a pair of color-filtered stereo images in a single shot and then estimates the disparity by registrating the pair of color images [7,8]. A salient region-based local disparity extraction method was proposed for robust cross-channel disparity estimation using phase correlation matching (PCM) in [9]. However, it cannot successfully register the pair of different color images since the PCM was originally devised to match the same color or monochrome images [10]. To solve this problem, Holloway et al. proposed the cross-channel normalized gradient (CCNG) [11]. The feature-based matching strategy is not sensitive to intensity difference, which make cross-channel disparity estimation robust. However, the estimated disparity becomes inaccurate in the weak feature regions.

In this paper, novel cross-channel disparity estimation and color registration methods are presented. To solve Holloway’s cross-channel disparity problem [11], the proposed disparity estimation method uses two features such as edge and texture for robust disparity estimation in the cross-channel condition. In addition, the adaptive weight cost function based on the distance transform (DT) is proposed to improve the accuracy of the estimated disparity in the weak feature regions. For color registration, the color mapping matrix (CMM) based on superpixel segmentation is proposed. As a result, the proposed method provides a finer disparity edge and more accurate disparity value at the weak feature regions. Finally, a high-quality image can be obtained.

## 2. Background

**Figure 1:**
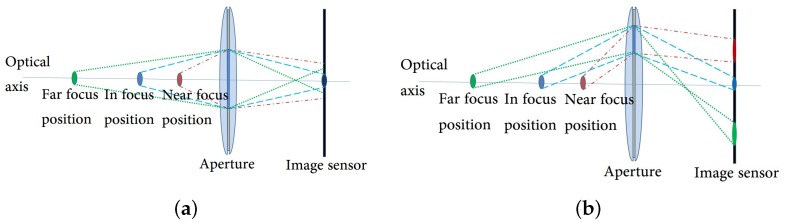
Imaging system with single aperture: (**a**) paths of light rays in a conventional optical system; (**b**) paths of light rays in a single off-axis aperture system.

A conventional camera has an aperture that is aligned on the optical axis. The light ray from a point of an input scene generates the corresponding convergence pattern on the image sensor. The shape of the convergence pattern may be either a point or a circle, whose size is determined by the distance of the object from the camera, as shown in Figure 1a. If the aperture deviates from the optical axis as shown in Figure 1b, the convergence point moves away from the image center. In this configuration, the convergence point on the image sensor moves up and down depending on the distance of the object from the camera.

**Figure 2:**
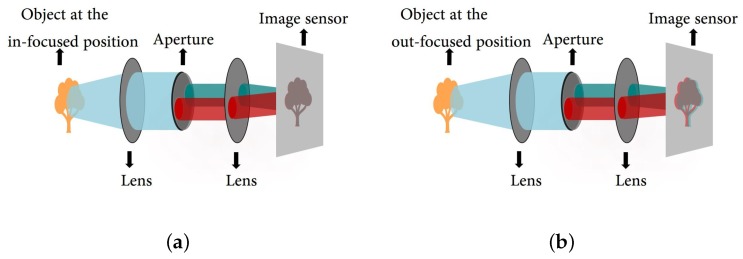
The dual color-filtered aperture (DCA) system: (**a**) the DCA configuration with an object at the in-focus position; and (**b**) the DCA configuration with an object at the out-of-focus position.

Figure 2 shows the optical system in the DCA system. Two off-axis apertures with a pair of complementary color filters (e.g., red and cyan) are deployed in the system. The DCA optical system generates the same image of the conventional camera system in the in-focus region, but generates a color-misaligned image in the out-of-focus region.

**Figure 3:**
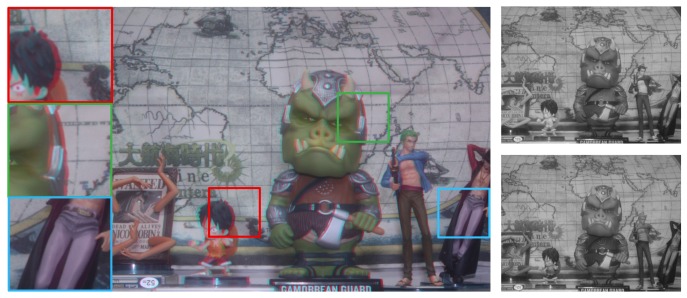
**Left**: Acquired image using the DCA camera; **upper right**: the red channel image; and **lower right**: the green channel image.

An acquired image by the DCA camera is shown on the left side of Figure 3. We used the same color filters shown in Figure 2. Color misalignments between red and green channels are produced according to the distance of objects from the in-focus position of the camera, which are shown in three regions of the red, green and blue boxes. Therefore, disparity information can be obtained from the DCA image by calculating the amount of misalignment between color channels. However, intensity difference issues need to be solved for cross-channel disparity estimation, as shown on the right side of Figure 3.

## 3. Cross-Channel Disparity Estimation and Color Channel Alignment

**Figure 4:**
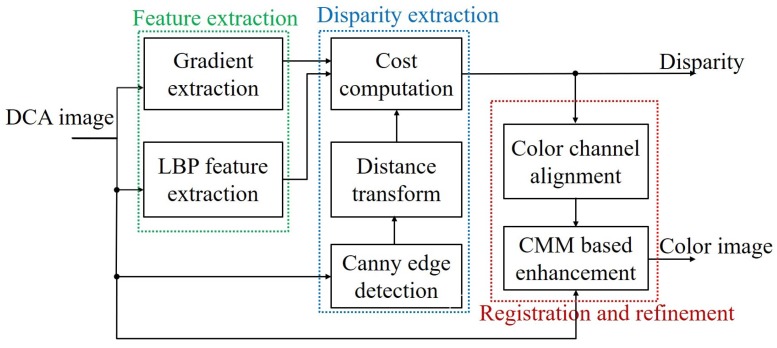
Block diagram of the proposed system. CMM, color mapping matrix.

**Figure 5:**
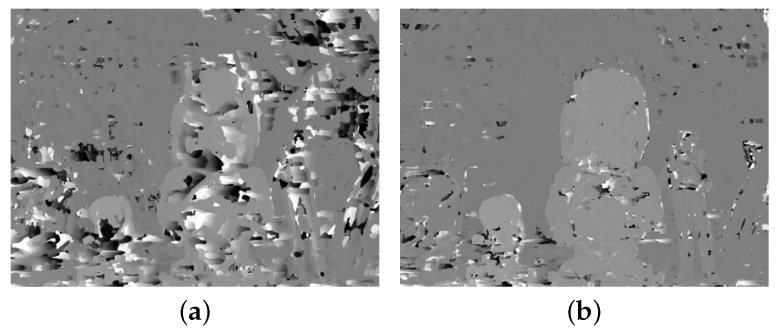
Poor disparity estimation results of a DCA image using the traditional method: (**a**,**b**) are matching results using sum of absolute differences (SAD) and normalized cross-correlation (NCC), respectively.

The block diagram of the proposed method is shown in Figure 4, which consists of three parts: (i) feature extraction; (ii) disparity extraction; and (iii) registration and refinement. The details of each part are described below. The DCA used in this paper has red and cyan filters, where the red channel is used as the reference image and the cyan channel is the target image. However, the main problem of the DCA system is the disparity estimation among different color channels, as shown in the right images of Figure 3. Since it is difficult to match important features among different color channels, traditional matching algorithms, such as sum of absolute differences (SAD) and normalized cross-correlation (NCC), exhibit poor performance in estimating the cross-channel disparity, as shown in Figure 5a,b, which are generated using red and green channels [12,13,14].

### 3.1. Feature Extraction

**Figure 6:**
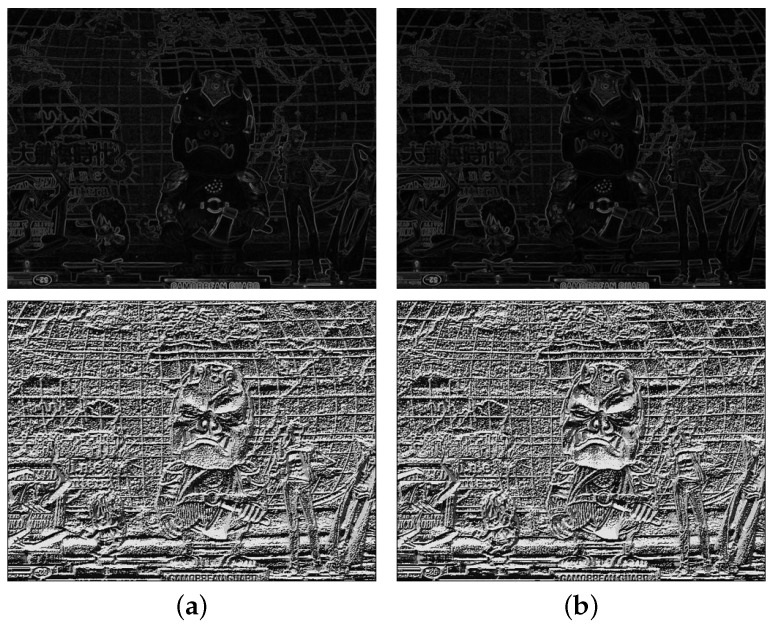
The gradient magnitude and LBP images of channels shown in Figure 3: gradient magnitude (**top**); LBP (**bottom**) of (**a**) red and (**b**) green channels.

Inspired by Holloway’s CCNG, the gradient and local binary pattern (LBP) feature values are combined to generate disparity map since the gradient discontinuity is preserved between different color channels. Figure 6 shows that edges are preserved among color channels with different intensities, whereas the matching error increases at the weak feature regions.

Ahonen et al. observed that the LBP generates a local texture pattern that is robust against illumination difference [15]. The LBP feature is expressed as:

(1) $$L\left(u,v\right)=\sum_{n=0}^{N-1}2^{n}\cdot \Lambda (\eta_{n}-\eta_{c}),$$

where *c* represents the central pixel (u,v) and *n* the index of neighborhood pixels centered on *c*. $\eta_{c}$ and $\eta_{n}$ represent the central pixel and neighborhood pixel, respectively. *N* is the total number of neighborhood pixels, and Λ(k) is a function that returns either one if k≥0 or zero, otherwise. Figure 6 shows the LBP features of the different color channel images for N=8. Since all the cross-channel LBP images show alike patterns including flat regions, it can be used for cross-channel similarity measurement.

### 3.2. Disparity Extraction

**Figure 7:**
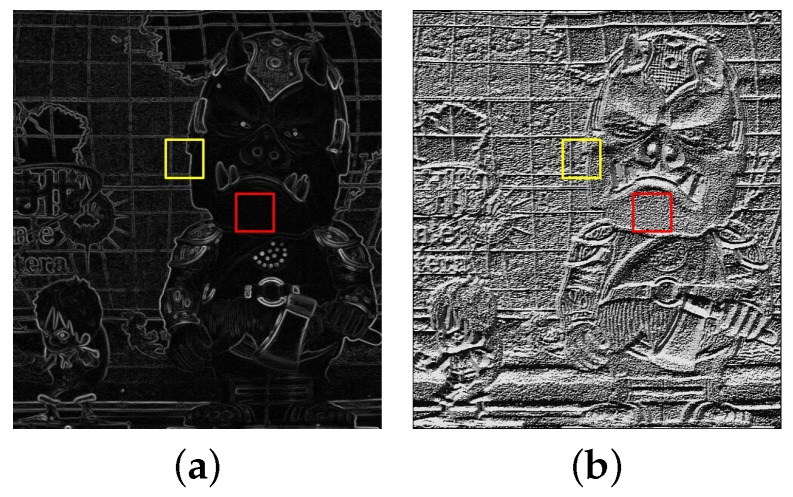
Comparison of the gradient magnitude and LBP feature: (**a**) two blocks selected in gradient magnitude image and (**b**) LBP feature image.

Figure 7 shows the local magnification of gradient magnitude and LBP for the red channel; the yellow block contains the strong gradient value and affluent LBP feature, which is beneficial for block matching; while in the red block, the gradient magnitude feature is extremely weak, which gives less of a contribution to block matching. What is more, this indistinct gradient information may lead to ambiguity when the gradient feature and LBP feature are combined in disparity estimation.

To solve the above problem, an adaptive weight objective function is proposed to coordinate the contribution of the gradient component and the LBP component in different regions for disparity extraction. The proposed objective function is defined as:

(2) $$C\left(d\right)=\sqrt{\sum_{\left(u,v)\in w(p\right)}\left[1-W\left(u,v\right)\right]G_{R}\left(u,v\right)G_{T}\left(u+d,v\right)+\sum_{\left(u,v)\in w(p\right)}W\left(u,v\right)L_{R}\left(u,v\right)L_{T}\left(u+d,v\right)},$$

where *p* represents the pixel index of the image, *d* the disparity in the search range [-s,s], w(p) the set of pixels centered at *p* indicating the selected block for matching, (u,v) the two-dimensional pixel coordinate, *G* the gradient magnitude, *L* the LBP feature value and subscripts *R* and *T*, respectively, the reference and target images.

The weight W(u,v) is generated with the edge location using DT. Given a binary image with two sets of pixels:

(3) $$\begin{array}{c} S=set\;of\;0\;,\;the\;non-edge\\ \bar{S}=set\;of\;1,\;the\;edge \end{array},$$

the production of DT for each pixel (u,v)∈S is assigned by following:

(4) $$W\left(u,v\right)=\min\left(\sqrt{{\left(u-i\right)}^{2}+{\left(v-j\right)}^{2}}\right),$$

where (i,j) is an element of $\bar{S}$, and each pixel value of the non-edge region is assigned by the minimum distance with the edge region.

**Figure 8:**
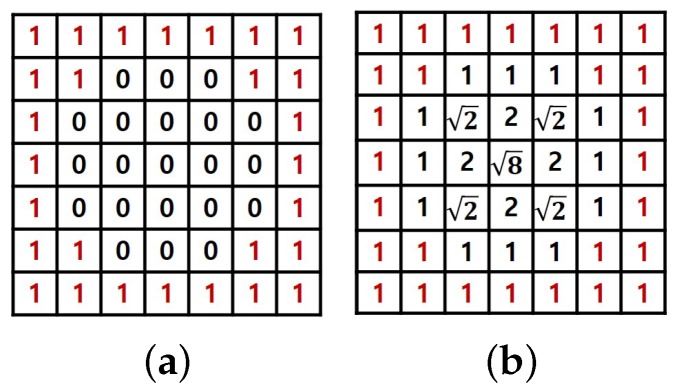
Process of the distance transform (DT): (**a**) binary image and (**b**) the result of DT.

The process of DT is simulated in Figure 8. With the Canny edge image as binary input, in Equation (2), the weight generated by DT can make the LBP component contribute more at the flat regions and the gradient component contribute more at the edge regions. The weight W(u,v) is normalized to [0,1] for convenient calculation of Equation (2). Compared with the single-feature-based CCNG, Equation (2) integrates more comprehensive feature information with the adaptive weight coefficient.

To reduce instability in computing the differences of cross-channel gradient magnitude and LBP value, block normalization is applied to both quantities as:

(5) $${\bar{G}}_{\alpha }\left(u,v\right)=\frac{G_{\alpha }\left(u,v\right)}{{∥G_{\alpha }∥}_{2}}\;\;,$$

(6) $${\bar{L}}_{\alpha }\left(u,v\right)=\frac{L_{\alpha }\left(u,v\right)}{{∥L_{\alpha }∥}_{2}}\;\;,\alpha \in \left\{R,T\right\},$$

where ${\bar{G}}_{\alpha }$ and ${\bar{L}}_{\alpha }$ are used to substitute the gradient and LBP component in Equation (2), respectively.

The disparity for every pixel location *p* can be determined by maximizing Equation (2), which yields the disparity map as:

(7) $$D\left(p\right)=\underset{d}{\arg\max}\left(C\left(d\right)\right),$$

**Figure 9:**
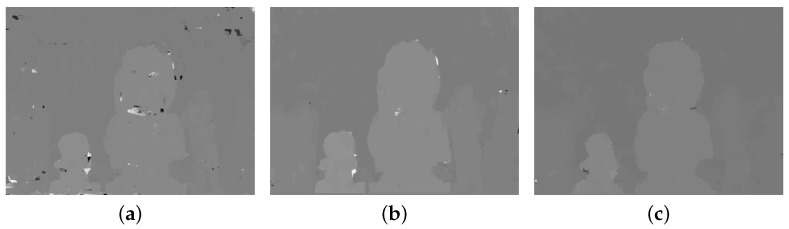
Disparity map comparison: (**a**) disparity generated by cross-channel normalized gradient (CCNG); (**b**) disparity generated by our method with a constant weight matrix, whose pixels value is assigned as 0.5; and (**c**) disparity generated by our method with adaptive weight.

Figure 9 shows the disparity comparison with single-feature-based CCNG and the disparity improvement by the adaptive weight coefficient. Comparing Figure 9a,b, the proposed method shows an apparent improvement over the single-feature-based CCNG in the flat regions. Comparing Figure 9b,c, the adaptive weight strategy can further reduce the disparity error in the flat regions and lead to a finer disparity boundary according to the object shape.

### 3.3. Registration and Refinement

Given the disparity prior, cross-channel image registration is performed by generating a new reference channel that is aligned with two target channels. Firstly, generate the initially aligned reference channel using estimated disparity map. Secondly, the CMM is generated to refine each local segment of the image. More details of each step are described below.

**Figure 10:**
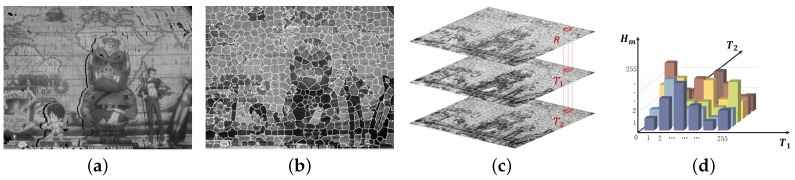
Segmentation process of the DCA image and CMM: (**a**) initially aligned reference channel *R* using the estimated disparity; (**b**) a segmented target channel using superpixels; (**c**) segmentation results of all three channels; and (**d**) the segment-wise CMM.

The input reference channel is initially aligned by pixel-wise shifting according to the estimated disparity map, as shown in Figure 10a. The initially aligned reference channel has missing pixels at some regions with occlusion, periodic texture and insufficient salient features. To refine the registration, one target channel $T_{1}$ is divided into segments, as shown in Figure 10b using SLIC superpixels [16], where the segmentation boundaries are applied to another target channel $T_{2}$ and initial aligned reference channel *R*, as shown in Figure 10c. Since most pixels in the same segment have a similar color, the missing intensity values of *R* can be recovered using pixels that belong to the same segment.

We propose a color mapping strategy by generating the CMM that represents the cross-channel intensity relationship of the segment. The CMM is generated using the following equation:

(8) $$H_{m}\left(T_{1}\left(x,y\right),T_{2}\left(x,y\right)\right)=R_{m}\left(x,y\right),\;if\;R_{m}\left(x,y\right)\neq 0,$$

where m∈[1,M] represents the segment index, *M* the total number of segments in the image and (x,y) the spatial coordinates in the *m*-th segment. Only pixels whose reference channel correspondence exists are chosen to structure the CMM. As shown in Figure 10d, intensity values of two target channels, denoted as $T_{1}\left(x,y\right)$ and $T_{2}\left(x,y\right)$, indicate the CMM index, and CMM value $H_{m}\left(T_{1}\left(x,y\right),T_{2}\left(x,y\right)\right)$ is assigned by the *m*-th initially aligned reference channel’s intensity value $R_{m}\left(x,y\right)$.

Given the CMM, the reference channel is enhanced using the following equation:

(9) $${\hat{R}}_{m}\left(x,y\right)=\left\{\begin{array}{c} H_{m}\left(T_{1}\left(x,y\right),T_{2}\left(x,y\right)\right),\;if\;R_{m}\left(x,y\right)=0\\ R_{m}\left(x,y\right),\;\;\;\;\;\;\;\;\;\;\;\;\;\;\;\;\;\;otherwise \end{array}\right.,$$

where ${\hat{R}}_{m}$ represents the *m*-th segment of the enhanced reference channel $\hat{R}$. Missing pixels in *R* are recovered by the CMM value with the target channel’s intensity according to the corresponding matrix index.

**Figure 11:**
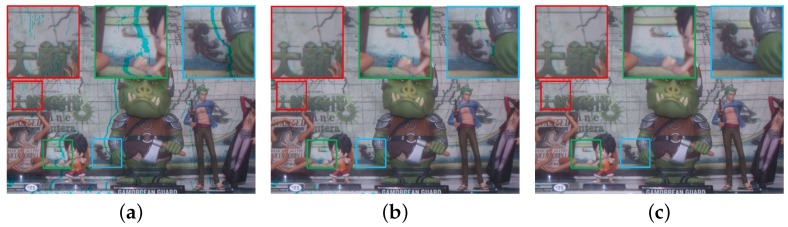
Comparison of color registration results: (**a**–**c**) registration using the initially aligned reference image, refinement with CMM, and refinement with enhanced CMM.

Figure 11a shows the result of color registration with the initial aligned reference channel. By comparing with Figure 11b, the small-scale missing pixels of the red box were well recovered, which was caused by the disparity error. However, the large-scale missing pixels, shown in green and blue boxes, were not completely recovered. The missing pixels observed at the two boxes were caused by occlusion between the cross-channel stereo image. The large chunk of missing pixels in the initially aligned reference channel make the CMM unreliable. To solve this problem, a simple matrix filling strategy is applied. Figure 11c shows the enhanced cross-channel color registration result.

**Figure 12:**
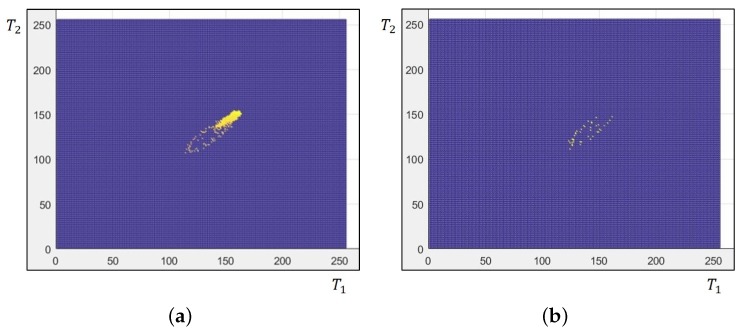
Color mapping matrices: (**a**) CMM and (**b**) local points without corresponding pixels in the initially aligned reference channel.

Figure 12 visualizes matrices to explain the registration enhancement process. Figure 12a shows the CMM using the Cartesian coordinate generated from the image block where missing pixels exist. The yellow dots represent the local points where value exists in the matrix. According to Equation (8), the x and y axes of the coordinate represent the intensity value. The outfit of visualized CMM looks similar to the 2D histogram of $T_{1}$ and $T_{2}$. However, the CMM has only values of aligned *R* without missing pixels, as shown in Figure 12a. Therefore, the values for missing pixels are shown in Figure 12b by extracting points existing only in the 2D histogram.

A fully-cross-channel color mapping matrix can be generated by filling the points of missing pixels as shown in Figure 12b with the nearest CMM value, whose location is shown in Figure 12a. The four-neighborhood strategy is employed in the nearest CMM value search. The registration result with enhanced CMM completely recovers non-value regions. As shown in Figure 11c, the registration gives a significant improvement with the full CMM, and the large-scale missing pixels can be recovered.

## 4. Experimental Results

**Figure 13:**
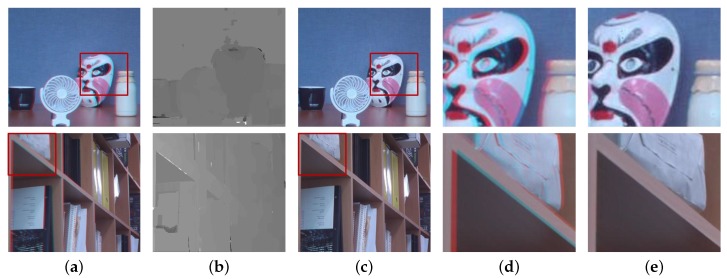
Proposed cross-channel disparity estimation and registration results. (**a**) Input images, (**b**) disparity maps, (**c**) registered images and (**d**,**e**) magnified regions of (**a**,**c**), respectively.

This section presents experimental results of the proposed methods and comparison with existing methods. Figure 13 shows the results of the proposed cross-channel disparity estimation and registration. The input images shown in Figure 13a are captured images by the DCA camera, where red and cyan color filters are applied on the aperture. The experimental results show that the proposed adaptively weighted cost function and color mapping strategy could generate good disparity maps in the cross-channel condition and correct misaligned color channel images, as shown in Figure 13b,c, respectively. As shown in Figure 13d,e, the proposed registration and refinement process could generate naturally aligned color images by recovering the missing pixels due to occlusion and disparity error.

**Figure 14:**
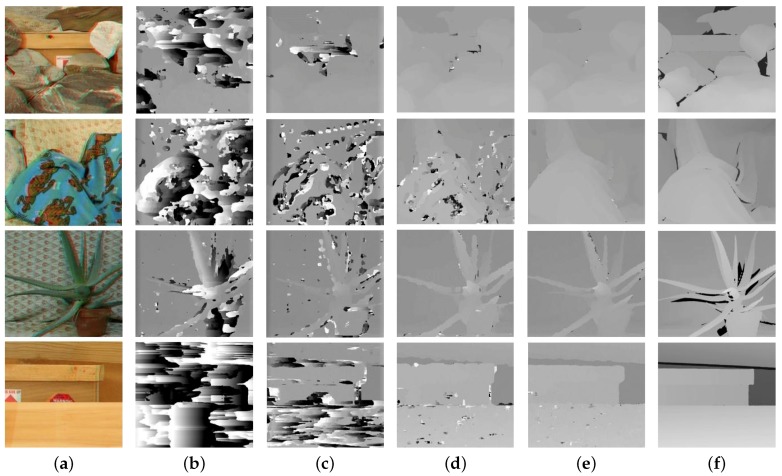
Disparity extraction of various matching methods with Middlebury stereo images ‘Rocks’, ‘Cloth’, ‘Aloe’ and ‘Wood’ [17]. (**a**–**f**) Input DCA image, disparity with SAD, NCC, Holloway’s CCNG, our method and the ground truth disparity.

For objective evaluation of the experimental results, the Middlebury stereo image dataset was used [17], and the performance of the proposed method was compared with existing methods. All DCA images were generated from stereo images, whose red channel played the role of the left image and blue and green channels the right image. The proposed method was compared with well-known matching methods SAD, NCC and Holloway’s CCNG. The disparity estimation results with various methods are shown in Figure 14. We applied same parameters to all images for performance evaluation. For disparity estimation parameter, all the images had a unified image size of 552 × 495, and the block size and search range are adopted as 20 × 20 and 1.5-times the block size respectively. For registration parameter, superpixel segment is set as M=800.

As shown in Figure 14, intensity difference-based SAD produced the worst disparity result, especially for a scene without distinct texture differences, like ‘Wood’. NCC had slight improvement with normalization, while the result was still unacceptable. Feature-based CCNG and the proposed method had significant improvement compared with SAD and NCC. The CCNG’s result was not good due to the insufficiency of a single feature, and the proposed method led to more accurate disparity in the weak-feature regions by combining features and a finer disparity edge value using the adaptive weight coefficient strategy. With the ground truth in the Middlebury stereo dataset, the disparity quantitative evaluation can be done by the following measure:

(10) $$Error\;rate=\frac{\sum_{(x,y)\in D\cap GT}(|D\left(x,y\right)-GT\left(x,y\right)|)>\delta }{N_{D\cap GT}},$$

where GT represents the ground truth disparity map, $N_{D\cap GT}$ is the number of the pixels that have values in the disparity map and the ground truth map simultaneously and δ is the error tolerance. The values of δ were set as one and two, and the error rate is summarized in Table 1 and Table 2. The disparity generated by SAD and NCC gave a high error rate, which means that intensity-based methods were limited in cross-channel matching. CCNG had a lower error rate than SAD and NCC, while the accuracy was degraded in a sparse gradient scene like ‘Cloth’ and ‘Wood’. As expected, by combining gradient and LBP features, the proposed method gave the lowest error rate in all the scenes.

**Figure 15:**
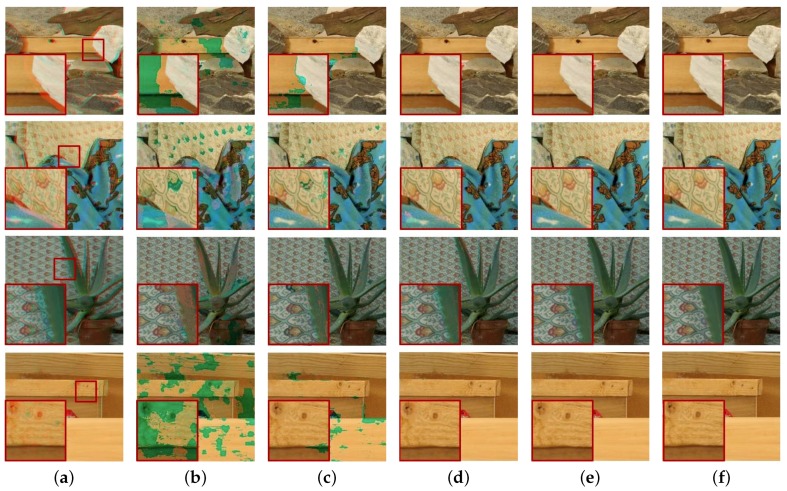
Color registration with the corresponding disparity prior shown in Figure 14 by using the proposed cross-channel registration and refinement strategy. (**a**) input DCA image, (**b**) registration by SAD, (**c**) NCC, (**d**) Holloway’s CCNG, (**e**) the proposed method and (**f**) the ground truth color image.

With the proposed cross-channel registration method, all the disparity maps generated with various methods were applied to aligning color channels. the corresponding cross-channel image registration results are shown in Figure 15. The original stereo single-view image can be considered as the ideal cross-channel registration result, as shown in Figure 15e. For objective evaluation of the registration quality, peak-to-peak signal-to-noise ratio (PSNR) and structural similarity measure (SSIM) were calculated [18].

A higher PSNR indicates a lower noise image, and a higher SSIM indicates higher similarity between registration and original single-view images. As shown in Table 3, the incorrect disparity by SAD and NCC yielded unreliable color registration. The PSNR and SSIM values of the proposed method were higher than the others. As shown in Table 1 and Table 2, the disparity error rate of CCNG was higher than the proposed method, while the PSNR and SSIM values shown in Table 3 were similar to the proposed method, which means that the proposed registration strategy could generate a decent color registration with acceptable disparity, which was not intact.

## 5. Conclusions

In this paper, we presented a cross-channel disparity extraction and color registration method with an adaptive weight cost function based on the feature. The robustness of the proposed method benefits from the sufficient feature information, and the disparity accuracy is further improved by the adaptive weight coefficient. After color channel registration using the disparity prior, a novel CMM image refinement strategy is proposed for enhancing the color image. Even if the disparity prior has a slight defect, image refinement can bring out a decent registration. As shown in the experimental results, the proposed method outperforms conventional methods in disparity estimation and generates high-quality image registration. Assuming that most of the images are captured by a conventional image sensor, the proposed disparity estimation method cannot perform well at a certain region where the color is one of the pure colors of the Bayer filter mosaic. This case is an inveterate problem for cross-channel disparity estimation since feature elements exist in one of three color channel images only. Studies of the relationship between the color channel and robust features across color channels are considered for future study. The proposed method can be used for disparity estimation in 3D scene reconstruction and intelligent driver assistance systems. 

References yes

## Figures and Tables

**Table 1:** Quantitative disparity evaluation with ground truth (error tolerance = 1).

Error Rate	SAD	NCC	CCNG	Proposed Method
Rocks	0.8167	0.5459	0.3388	0.1832
Cloth	0.9060	0.7374	0.4900	0.3571
Aloe	0.6057	0.5128	0.2124	0.2029
Wood	0.9901	0.7062	0.2109	0.1843

**Table 2:** Quantitative disparity evaluation with ground truth (error tolerance = 2).

Error Rate	SAD	NCC	CCNG	Proposed Method
Rocks	0.7062	0.3878	0.0990	0.0407
Cloth	0.8280	0.5278	0.2506	0.0627
Aloe	0.4615	0.3259	0.1435	0.1111
Wood	0.9798	0.5171	0.1142	0.0599

**Table 3:** Quantitative registration comparison using PSNR and SSIM between our method and conventional algorithms.

		SAD	NCC	CCNG	Proposed Method
Rocks	PSNR	26.3083	30.8668	35.8874	35.9138
	SSIM	0.8990	0.9580	0.9784	0.9792
Cloth	PSNR	25.7239	29.8107	33.4656	35.6846
	SSIM	0.8257	0.8970	0.9496	0.9733
Aloe	PSNR	31.9656	34.3602	35.2166	36.0752
	SSIM	0.9275	0.9499	0.9654	0.9698
Wood	PSNR	20.9945	25.1666	40.9911	41.7191
	SSIM	0.7902	0.8908	0.9892	0.9904
